# Genomic surveillance of invasive Streptococcus pneumoniae strains in south Tunisia during 2012–2022

**DOI:** 10.1099/mgen.0.001448

**Published:** 2025-07-17

**Authors:** Fahmi Smaoui, Boutheina Ksibi, NourElhouda Ben Ayed, Sonia Ktari, Omar Gargouri, Senda Mezghani, Basma Mnif, Faouzia Mahjoubi, Héla Karray, Adnene Hammami

**Affiliations:** 1 Research Laboratory Microorganisms and Human Disease “MPH LR03SP03”, Laboratory of Microbiology, Habib Bourguiba University Hospital, Sfax, Tunisia; 2 Laboratory of Microbiology, Faculty of Medicine of Sfax, University of Sfax, Sfax, Tunisia

**Keywords:** genomic surveillance, global pneumococcal sequence cluster, invasive pneumococcal disease, *Streptococcus pneumoniae*, Tunisia, whole-genome sequencing

## Abstract

**Purpose.** Invasive pneumococcal disease (IPD) remains a major global public health concern due to its high morbidity and mortality rates, particularly among children and the elderly. This study aimed to apply whole-genome sequencing (WGS) to characterize *Streptococcus pneumoniae* strains responsible for IPD in south Tunisia, including serotype distribution, clonal relationship and antimicrobial resistance (AMR) profiles.

**Methods.** A total of 148 IPD *S. pneumoniae* isolates were collected from the microbiology laboratory at Habib Bourguiba University Hospital in Sfax, Tunisia, between 2012 and 2022. These isolates underwent WGS using Illumina technology. Bioinformatic analyses were performed to determine serotype distribution, sequence types (STs), Global Pneumococcal Sequence Clusters (GPSCs), phylogenetic relationships and AMR determinants.

**Results.** Twenty-six different serotypes were identified, with the most prevalent being 14 (18%), 3 (13%), 19A (12%) and 19F (11%). The isolates showed high genomic diversity, as they belonged to 32 GPSCs and 59 STs. The most common GPSCs were GPSC-6, GPSC-10 and GPSC-44, associated with serotypes 14, 19A and 19F, respectively. The most frequent STs were ST2918, ST179 and ST3772. The most common resistance genes were *erm*B (53%) and *tet*M (55%), which were linked to resistance against erythromycin and tetracycline, respectively. There was a considerable concordance between WGS-based and phenotypic resistance profiles for most tested antibiotics, with few major and very major errors for most antibiotics. Temporal analysis showed a decline in serotypes 19F and 9V throughout the study period, which was associated with slight decreases in GPSC-6 and GPSC-44, while serotype 19A and GPSC-10 sharply increased.

**Conclusion.** This study highlights the substantial genomic diversity, serotype distribution and high prevalence of AMR among IPD *S. pneumoniae* isolates in south Tunisia, underscoring the need for continued surveillance and effective vaccination strategies to combat this persistent public health threat.

**Impact Statement**

*Streptococcus pneumoniae* remains a significant public health threat due to its ability to cause invasive pneumococcal disease (IPD), such as meningitis and sepsis, which result in considerable morbidity and mortality. The rise of antimicrobial resistance (AMR) in *S. pneumoniae* further complicates treatment strategies, highlighting the critical need for continuous monitoring of its genomic diversity, serotype distribution and resistance patterns. This study presents the first genomic analysis of IPD *S. pneumoniae* strains in Tunisia, revealing high genetic diversity and widespread prevalence of AMR. Over the past decade, notable changes were observed in the genetic make-up and serotype distribution of *S. pneumoniae*, including a decline in lineages associated with vaccine-targeted serotypes. However, the emergence of the GPSC-10 lineage, characterized by high AMR, underscores the urgent need for enhanced surveillance and the implementation of more effective vaccination strategies to limit the spread of resistant *S. pneumoniae* and address the growing challenge of AMR in the region.

## Data Summary

The sequencing data generated in this study have been deposited in the NCBI BioProject database under accession number PRJNA1182109. The associated Sequence Read Archive, BioSample and Genbank accession numbers are provided in Table S1. Additionally, all genome assemblies generated in this study are available via Zenodo at DOI: 10.5281/zenodo.15543227. Five tables and four figures are provided as supplementary materials. All supporting data, code and protocols have been provided within the article or through supplementary data files.

## Introduction

*Streptococcus pneumoniae* is an opportunistic pathogen that poses significant challenges to public health. It is responsible for a broad spectrum of diseases ranging from mild respiratory infections to severe and life-threatening conditions known as invasive pneumococcal diseases (IPD), including meningitis and bacteraemia [1,2]. These infections predominantly affect vulnerable populations, including young children, the elderly and individuals with compromised immune systems [3,6]. Among these groups, the impact of IPD can be particularly severe, often resulting in high morbidity and mortality rates [7].

The primary virulence factor of *S. pneumoniae* is its polysaccharide capsule, which is implicated in disease severity [8,9]. The pneumococcal capsule is characterized by high diversity, with over 100 different serotypes being identified [10]. Such diversity is crucial in enabling *S. pneumoniae* to persist in human populations and evade immune response. This complicates prevention efforts and poses challenges for vaccine development and public health strategies.

In the last decades, the treatment of pneumococcal infections has become increasingly challenging due to the growing problem of antibiotic resistance [11,14]. This rise in resistance has led to *S. pneumoniae* being recognized by the World Health Organization as a priority pathogen [15]. The widespread use of antimicrobial drugs has likely contributed to this issue by providing a selective advantage to resistant strains [16].

In Tunisia, IPD remains a significant public health issue, contributing to high morbidity, mortality and healthcare costs. Pneumococcal meningitis alone affects around 26 children under the age of 15 per million, resulting in 69 hospitalizations and 10 deaths annually [17]. To address this, Tunisian health authorities introduced pneumococcal conjugate vaccines (PCVs) in the private sector since 2008 and included PCV10 (GSK) in the national immunization programme since 2019 [18,19].

The ongoing surveillance of *S. pneumoniae* is essential to adapt vaccination and therapeutic strategies [20]. In recent years, whole-genome sequencing (WGS) has revolutionized the study of *S. pneumoniae*, providing detailed analysis of its genetic make-up, including serotype distribution, antibiotic resistance determinants and virulence factors. WGS also facilitates the tracking of *S. pneumoniae* clones and their transmission pathways, which is essential for understanding the epidemiology of IPD and developing effective public health strategies [21].

In this study, we conducted a comprehensive genomic investigation of *S. pneumoniae* strains responsible for IPD in south Tunisia between 2012 and 2022, focusing on serotype distribution, genetic diversity and antibiotic resistance determinants among the isolates.

## Methods

### Sample collection and serotyping

Between January 2012 and December 2022, a total of 201 non-duplicate *S. pneumoniae* isolates responsible for IPD were collected at the microbiology laboratory of Habib Bourguiba University Hospital in Sfax, Tunisia. Among these, 148 non-duplicate * S. pneumoniae* isolates were subjected to WGS. These isolates were collected from both paediatric (*n*=68) and adult patients (*n*=75) in south Tunisia, with age unknown for five patients. They were recovered from clinical specimens taken from sterile body sites, including blood (*n*=73), cerebrospinal fluid (CSF) (*n*=48) and other body fluids (*n*=27).

The study duration was subdivided into three distinct periods: period 1 (2012–2015), period 2 (2016–2019) and period 3 (2020–2022), the latter being shortly after the introduction of PCV10 (GSK) into the national immunization programme.

The identification of the isolates was confirmed using standard procedures, including Gram staining, optochin sensitivity and bile solubility tests. PCR targeting the autolysin (*lyt*A), pneumolysin (*ply*) and capsular (*cps*A) genes was conducted to validate the identification, as previously described [22,23]. Serotyping was performed using a combination of multiplex PCR [24] and latex agglutination (ImmuLex™ Pneumotest).

### Antimicrobial susceptibility testing

All isolates were tested for antimicrobial susceptibility for penicillin G, amoxicillin, cefotaxime and levofloxacin by measuring the minimum inhibitory concentration (MIC) using *E*-tests and for erythromycin (15 µg), gentamicin (500 µg), chloramphenicol (30 µg), tetracycline (30 µg), trimethoprim/sulfamethoxazole (1.25–23.75 µg) and rifampicin (5 µg) using the disc diffusion technique. The two methods were performed on Mueller–Hinton agar supplemented with 5% sheep blood and interpreted according to the recommendations of the Antibiogram Committee of the French Society of Microbiology (CA-SFM) and the European Committee on Antimicrobial Susceptibility Testing (EUCAST) guidelines. Results were categorically classified as ‘susceptible, standard dosing regimen’ (S), ‘susceptible, increased exposure’ (I) or ‘resistant’ (R). Both meningitis and non-meningitis breakpoints were applied for beta-lactams (Table S2, available in the online Supplementary Material). Multi-drug resistance (MDR) was defined as resistance to at least three classes of antibiotics. *S. pneumoniae* ATCC 49619 was used as a quality control strain.

### WGS and bioinformatic analysis

#### WGS, assembly and quality control

Genomic DNA was extracted from pneumococcal isolates through the QIAamp DNeasy blood and tissue method. Library preparation was performed using the NEBNEXT^®^ Ultra™ II DNA Preparation Kit. WGS was performed on the Illumina NovaSeq platform using 2×150 bp paired-end chemistry. Quality control and trimming of reads were performed with fastp software (v.0.23.4) [25]. The genome data were assembled with the Shovill (https://github.com/tseemann/shovill) wrapper (v.1.1.0). Assemblies were evaluated with the QUAST (v.5.0.2) tool to assess their quality [26].

#### *In silico* typing

Bioinformatic analysis was mostly performed following the Nextflow pipeline [27] developed by the Global Pneumococcal Sequencing (GPS) group (available at https://github.com/sanger-bentley-group/gps-pipeline). *In silico* serotyping was performed using seroBA (v.1.0.5) [28]. Sequence types (STs) of the *S. pneumoniae* isolates were determined by the sequences of seven housekeeping genes (*aroE*, *gdh*, *gki*, *recP*, *spi*, *xpt* and *ddl*) obtained from the results of WGS. Allelic numbers and STs were assigned using the mlst tool (v.2.23.0) (https://github.com/tseemann/mlst) and the PubMLST database (https://pubmlst.org/) for *S. pneumoniae*. Strains for which at least six of the seven alleles were identical were classified as belonging to a clonal complex (CC) using goeBURST (v.1.2.1) [29].

The genetic structure was further elucidated by assigning each isolate to a Global Pneumococcal Sequence Cluster (GPSC) using the k-mer-based clustering tool PopPUNK (v.2.6.0) [30] based on the GPS database (GPSC v6; *n*=42,163, available at https://www.pneumogen.net/gps/training_command_line.html) [21].

#### Detection of antimicrobial resistance determinants

Antibiotic resistance genes and mutations were detected from *S. pneumoniae* genomes using the custom Nextflow pipeline developed by the GPS group, which incorporates the ARIBA (v.2.14.6) tool [31] and the pipeline of the Centers for Disease Control and Prevention, available at https://github.com/BenJamesMetcalf/Spn_Scripts_Reference. In addition, penicillin-binding protein (PBP) allelic profiles were further checked using the PBPtyper (v.1.0.2) tool (https://github.com/rpetit3/pbptyper). A machine learning (ML) model based on PBP alleles was applied to predict beta-lactam resistance profiles [32]. The concordance between phenotypic and WGS-based predictions was assessed following the Food and Drug Administration recommendations, which define very major errors (predicted as susceptible but phenotypically resistant), major errors (predicted as resistant but phenotypically susceptible) and minor errors (any other discordance) [33].

#### Phylogenetic analysis

Core-genome SNP alignment was performed using the Snippy tool (v.4.6.0) (https://github.com/tseemann/snippy). Gubbins (v.3.2.1) [34] was employed to identify and remove recombination sites. A phylogenetic tree was constructed using IQ-TREE (v.2.0.3) [35], with the TMV+F+ASC+R2 substitution model, which was selected based on the Bayesian information criterion through ModelFinder [36]. Data were visualized with midpoint rooting using the Interactive Tree Of Life (iTOL) tool [37].

### Statistical analysis

Statistical analyses were conducted using the R language (v.4.4.0) through the R graphical user interface (R GUI). The Cochran–Armitage test for trend was employed via the DescTools library to analyse temporal changes in serotype distributions, pneumococcal lineages and resistance rates. A *P*-value threshold of 0.05 was set to determine statistical significance.

## Results

### Serotype distribution

The 148 invasive *S. pneumoniae* isolates were classified into 26 different serotypes using WGS and 25 serotypes using conventional techniques (PCR/latex agglutination). The WGS-based *in silico* serotyping method showed a high level of concordance with conventional methods, with a 96% (*n*=142/148 isolates) agreement rate. The remaining five cases showed partial discordance, with both *in silico* and conventional methods assigning them to the same serogroup (Table S3).

**Fig. 1.**
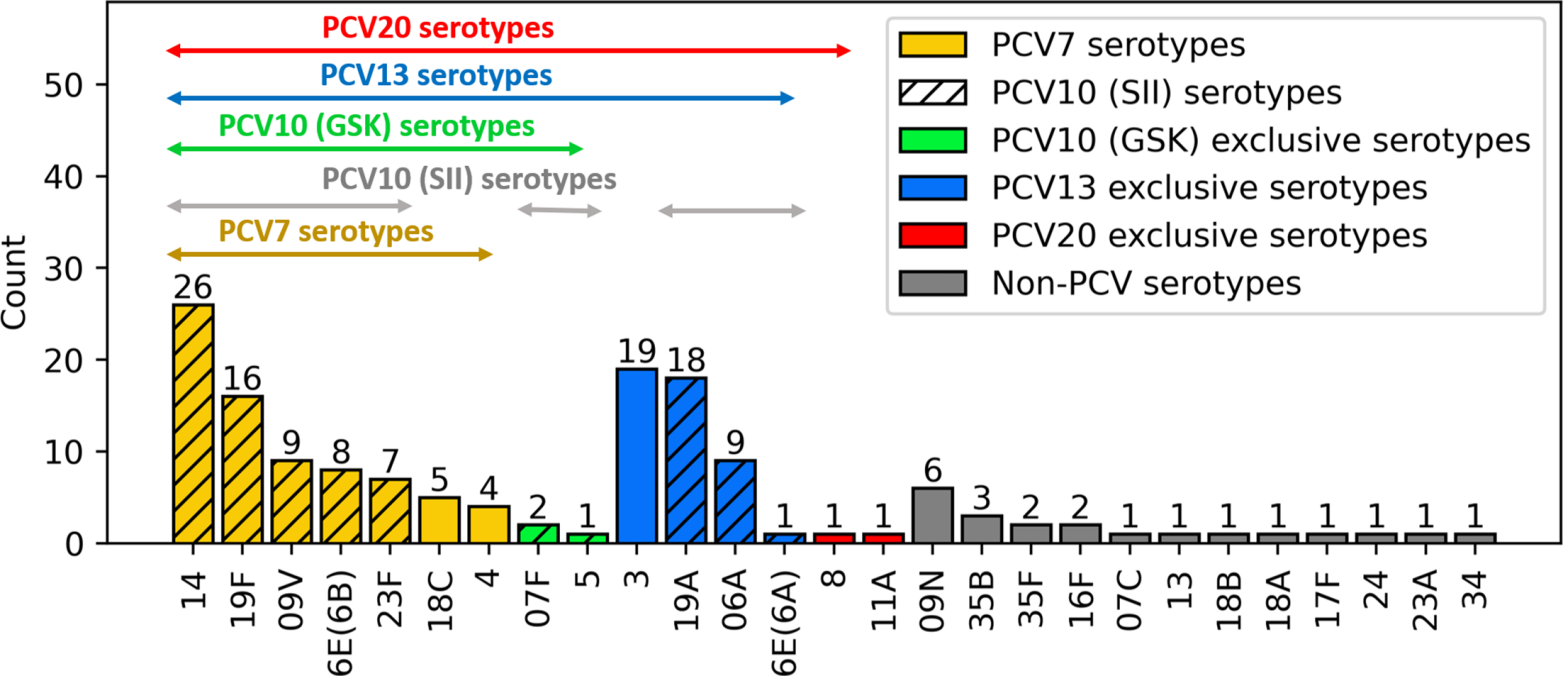
Distribution of *S. pneumoniae* serotypes according to their inclusion in PCVs in south Tunisia (2012–2022).

The most predominant serotypes based on WGS were 14 (18%), followed by 3 (13%), 19A (12%), 19F (11%), 6 A/6E(6A) (7%), 9V (6%) and 6E(6B) (5%) (Fig. 1). The theoretical vaccine coverage of the different PCVs was as follows: 51% for PCV7, 53% for PCV10 (GSK), 66% for PCV10 (SII), 84% for PCV13 and PCV15 and 86% for PCV20.

The plot visualizes serotypes as determined by WGS. Molecular type 6E(6A) was considered as a single serotype with 6A but was displayed separately for clarity.

### Antibiotic resistance profiles

#### Phenotypic resistance

Based on non-meningitis breakpoints, penicillin G resistance was observed in 6% of IPD *S. pneumoniae* isolates, amoxicillin resistance was noted in 12% of strains and none of the IPD isolates were resistant to cefotaxime. However, using meningitis breakpoints, 69% of strains were resistant to penicillin G, 48% to amoxicillin and 11.5% to cefotaxime.

**Fig. 2.**
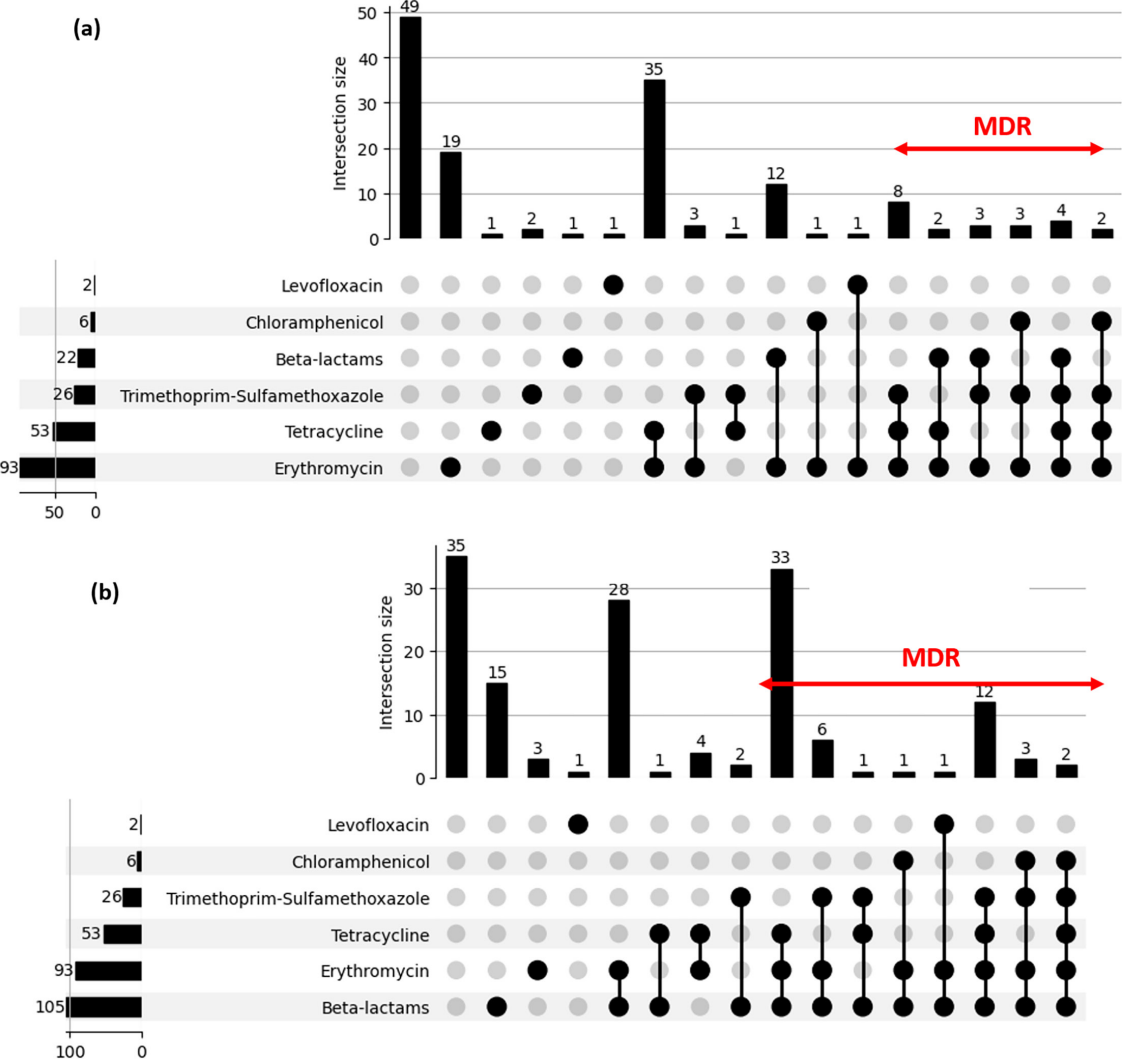
Upset plots of phenotypic resistance profiles based on non-meningitis breakpoints (**a**) and meningitis breakpoints (**b**).

The resistance rate to other antibiotics was observed as follows: erythromycin resistance was found in 63% of strains. Tetracycline resistance was detected in 36% of isolates. Resistance to trimethoprim/sulfamethoxazole was noted in 18% of cases. Chloramphenicol resistance was observed in 4% of strains. Levofloxacin resistance was rare, occurring in only two isolates (Fig. 2). All strains were susceptible to rifampicin and no high-level resistance was detected for gentamicin. MDR was observed in 15% of strains based on non-meningitis breakpoints. In contrast, 40% of isolates were classified as MDR when applying meningitis breakpoints, with 80% of MDR isolates exhibiting combined resistance to at least beta-lactams, erythromycin and tetracycline.

#### WGS-based resistance profiles

The 148 *S*. *pneumoniae* strains displayed significant diversity in PBP genes, with 23 previously reported *pbp*2A alleles, 27 *pbp*2B alleles and 20 *pbp*2X alleles. Additionally, 19 strains carried at least 1 newly identified PBP allele. ML predictions of MICs based on these alleles showed high specificity when predicting beta-lactam susceptibility, though the overall agreement was moderate with phenotypic resistance results of beta-lactams (Fig. S1). Most discordances were minor, except for three cases: one major discordance for amoxicillin (predicted as resistant but phenotypically susceptible), one major discordance for cefotaxime and one very major discordance for amoxicillin (predicted as susceptible but phenotypically resistant).

**Fig. 3.**
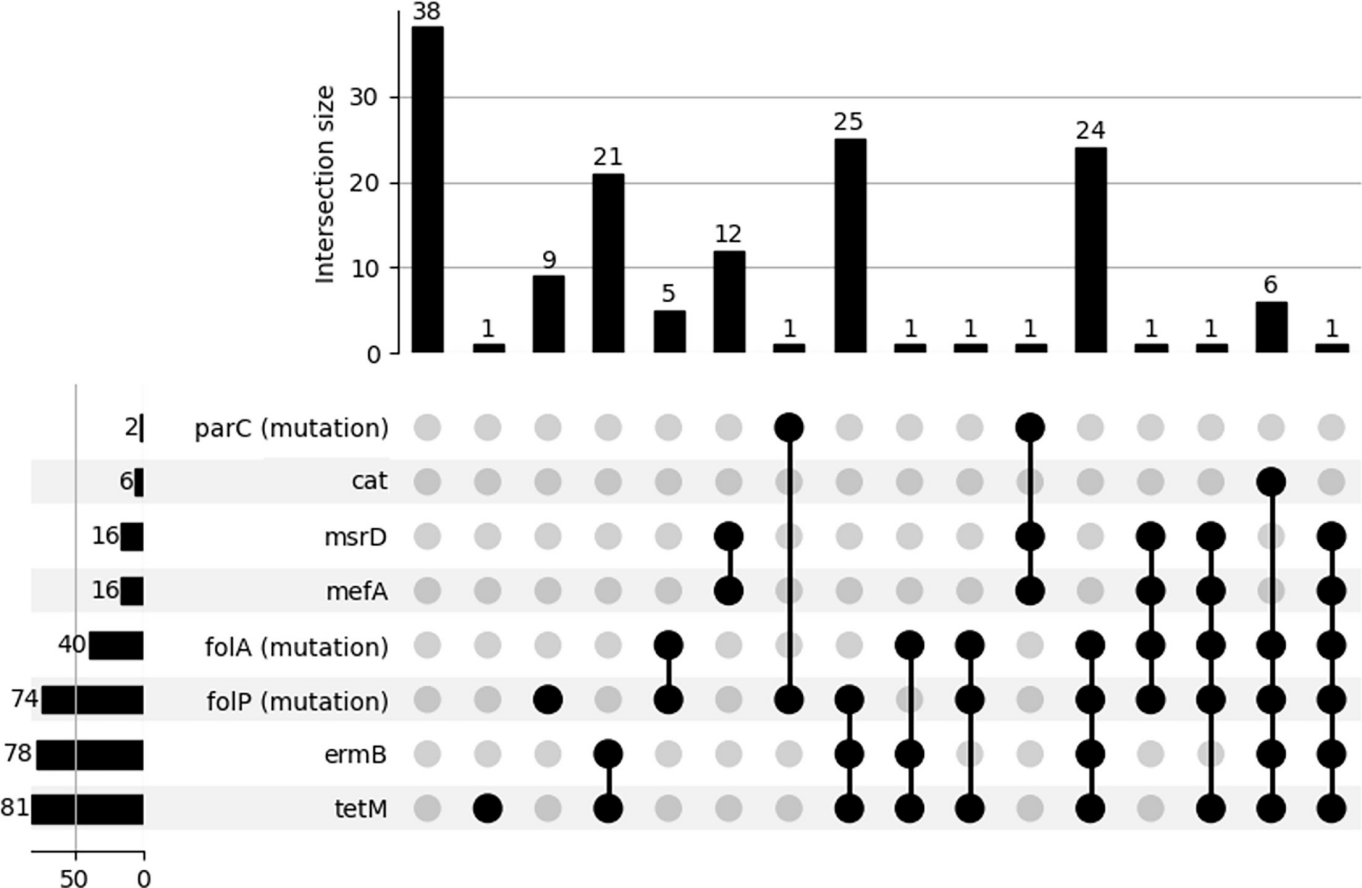
Upset plots of resistance determinant carriage based on WGS.

Regarding other antibiotic classes, the most frequently detected resistance genes were *tet*M, found in 55% of isolates, and *erm*B, found in 53% of isolates. In the majority of cases, both genes were detected together, except for three isolates that contained only *tet*M (Fig. 3). Sixteen (11%) strains harboured both *mef*A and *msr*D genes, and six (4.5%) strains carried the *cat* gene. The most common resistance mutations were observed within the *fol*A (27% of strains) and *fol*P (50% of strains) genes, while mutations in the *par*C gene were rare, with only two strains displaying the S79F and S79Y mutations.

The concordance analysis between antimicrobial resistance (AMR) determinants and phenotypic resistance revealed a perfect agreement for erythromycin and chloramphenicol (Fig. S2). All tetracycline-nonsusceptible (I+R) strains harboured the *tet*M gene, although it was also found in 24% of tetracycline-susceptible strains (major errors). The S79F and S79Y mutations in *par*C were exclusively linked to levofloxacin resistance. For trimethoprim-sulfamethoxazole, the concordance was relatively lower. All nonsusceptible strains carried mutations in *fol*A and/or *fol*P, with 88.5% (23 out of 26) of resistant strains having mutations in both genes. However, this combination of mutations (*fol*A and *fol*P) was also detected in 8% of susceptible cases (major errors).

### Pneumococcal lineages

Multilocus sequence typing of all *S. pneumoniae* strains revealed 59 distinct STs, including six novel STs (Table S4). Based on single-locus variations, these STs formed 13 CCs and 24 singletons. The most common STs were ST2918 (11%), ST179 (9.5%) and ST3772 (9.5%). Most STs (92.59%) were linked to a single serotype, while four STs were associated with two serotypes: ST63 (serotypes 14 and 23F), ST1381 (serotypes 18B and 18C), ST280 (serotypes 9V and 18C) and ST156 (serotypes 14 and 9V).

**Fig. 4.**
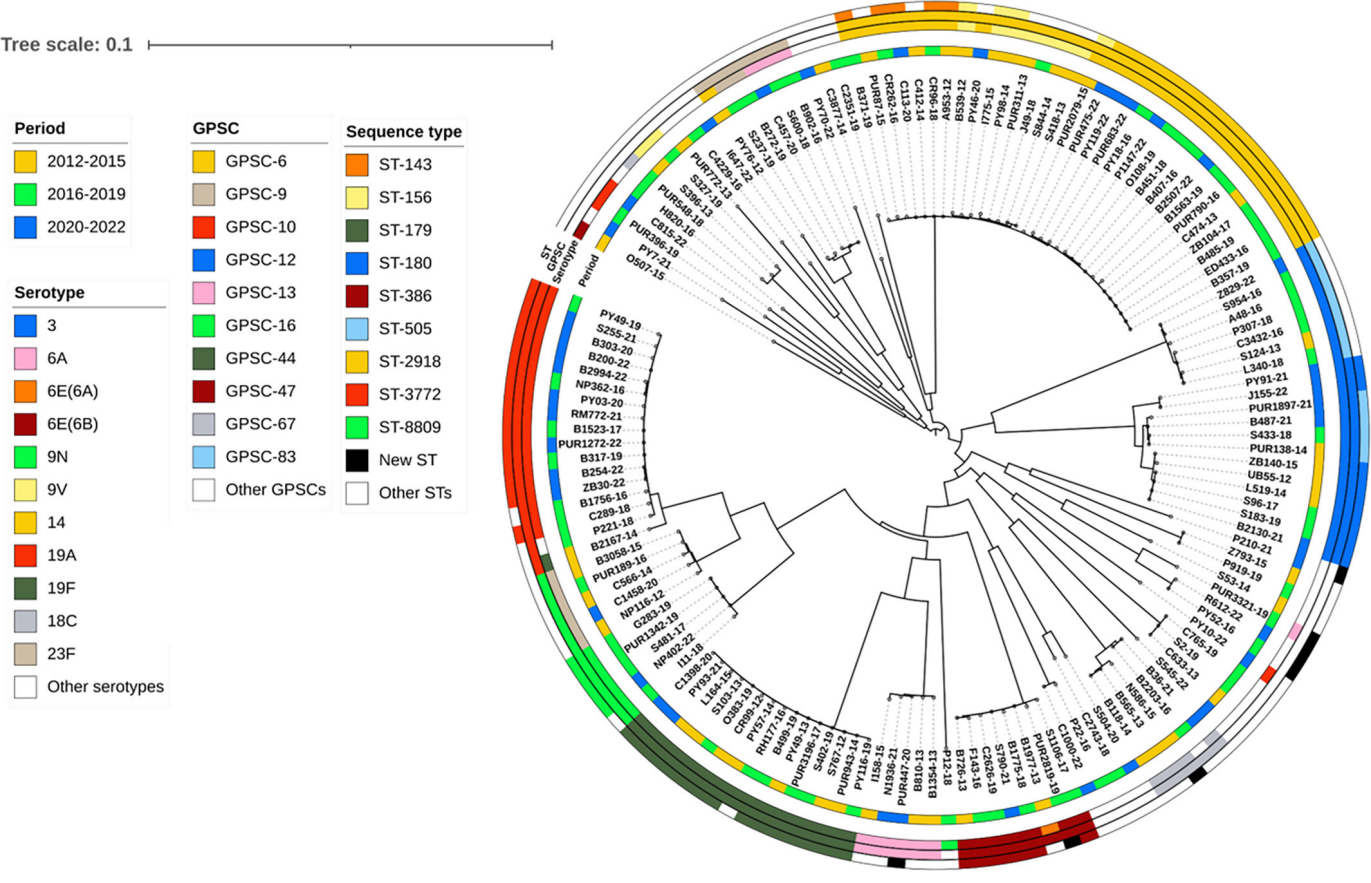
Phylogenetic tree of the 148 south Tunisian *S. pneumoniae* responsible for invasive pneumococcal diseases between 2012 and 2022.

Phylogenetic analysis revealed considerable genetic diversity of the 148 sequenced IPD *S. pneumoniae* isolates (Figs 4 and S3). PopPUNK clustering assigned these strains into 32 GPSCs. Within this classification, we identified one entirely new GPSC (GPSC-1124). The most common GPSCs were GPSC-6 (22%), GPSC-10 (11.5%), GPSC-44 (10%), GPSC-12 (8%) and GPSC-16 (7%). It is noteworthy that serotypes and STs were found to be correlated with the genome-based GPSCs (Table S5). Particularly, all sequences sharing the same ST were classified into a single GPSC. Furthermore, frequent serotypes (14, 19F, 19A and 6B) were predominantly associated with one GPSC (GPSC-6, GPSC-44, GPSC-10 and GPSC-47, respectively). On the other hand, serotype 3 isolates displayed notable genetic diversity and were assigned to GPSC-12 and GPSC-86 in 63% (*n*=12/19) and 37% (*n*=7/19) of cases, respectively. Also, serotype 6A isolates exhibited a high genetic diversity, being classified into GPSC-13 (*n*=5/10), GPSC-9 (*n*=3/10) and GPSC-213 (*n*=1/10), while the 6E(6A) isolate was classified into GPSC-47 (*n*=1/10).

**Fig. 5.**
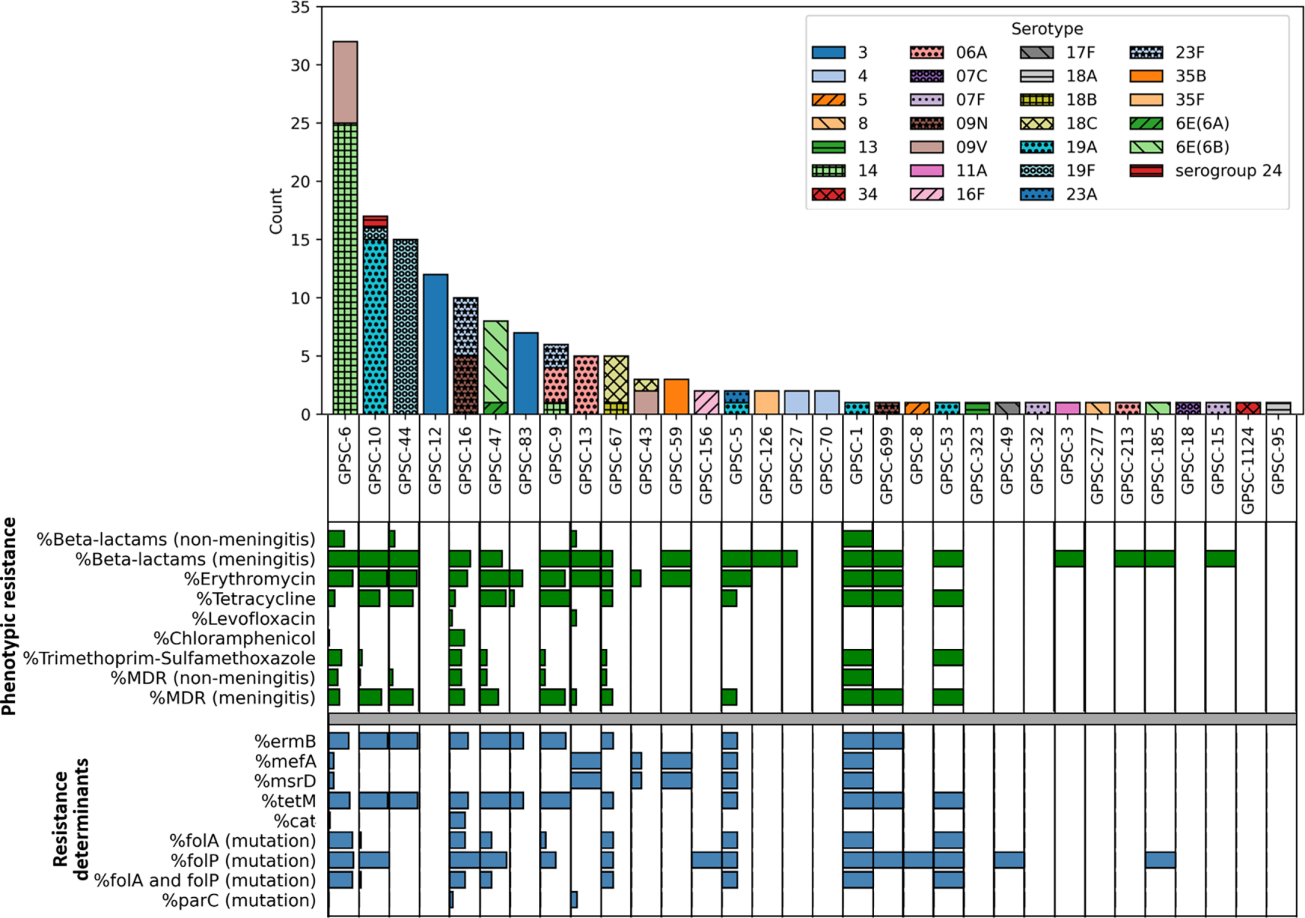
Distribution of phenotypic resistance and AMR determinants according to pneumococcal lineages.

Resistance rates varied considerably across GPSCs. Among the predominant GPSCs, GPSC-12 and GPSC-83, both associated with serotype 3, were largely susceptible to all tested antibiotics (Fig. 5). GPSC-12 strains lacked any resistance determinants, while three of the seven GPSC-83 isolates carried *tet*M and *erm*B. The GPSCs with the highest rates of MDR were GPSC-16 (40%), GPSC-6 (31%) and GPSC-47 (25%) when applying non-meningitis breakpoints. Based on meningitis breakpoints, MDR rates were high for GPSC-9 (83%), GPSC-44 (80%), GPSC-10 (76.5%) and GPSC-47 (62.5%). In contrast, the MDR rates for GPSC-16 and GPSC-6 were relatively lower, at around 50% and 37.5%, respectively.

### Dynamics of pneumococcal serotypes and lineages over time

**Fig. 6.**
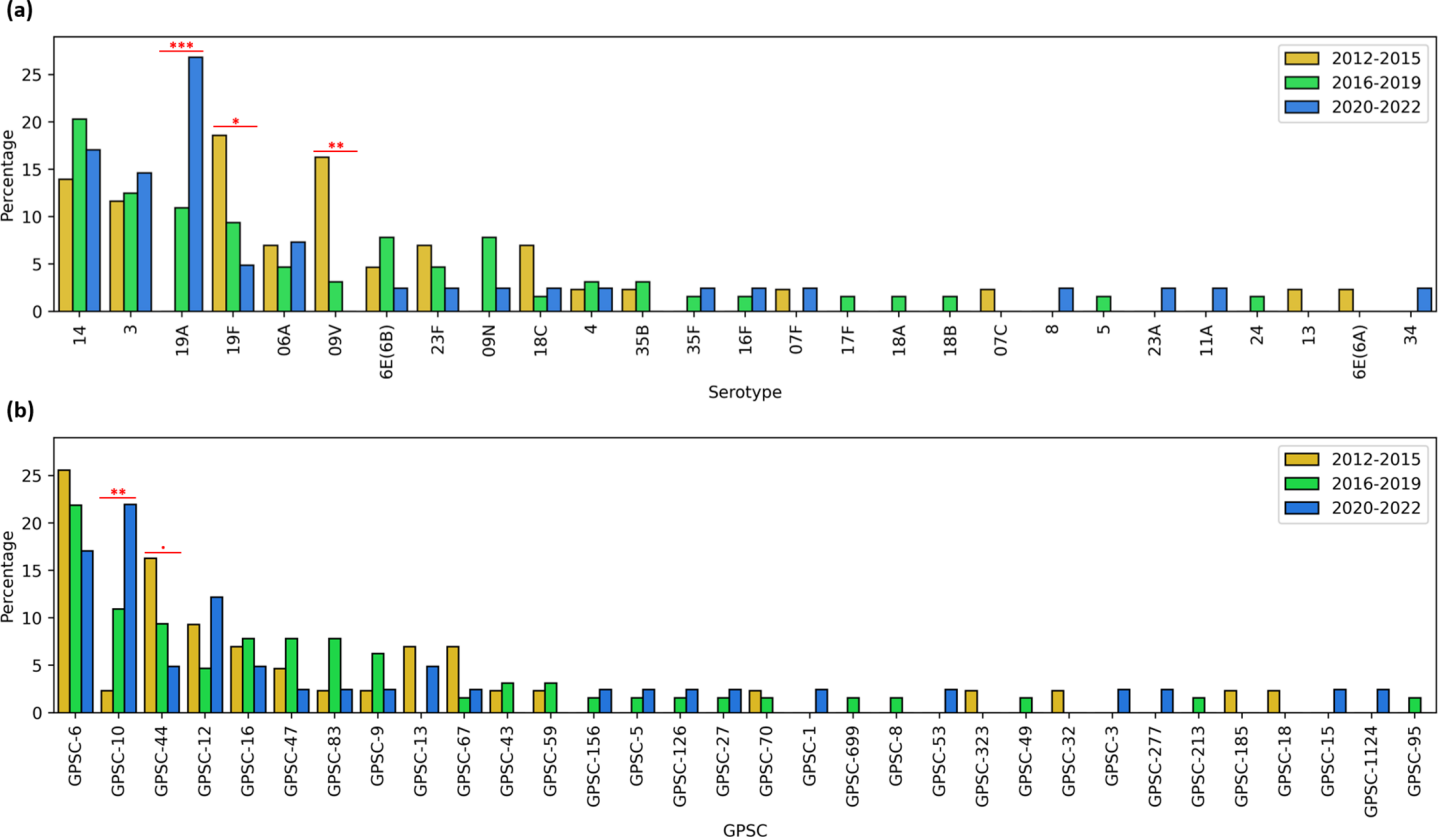
Evolution of *S. pneumoniae* serotype and GPSC distribution from 2012 to 2022 in south Tunisia. Statistical significance is indicated in red, with the following thresholds: *P*-value between 0.1 and 0.05 (‘.’), *P*-value between 0.05 and 0.01 (‘*’), *P*-value between 0.01 and 0.001 (‘**’) and *P*-value below 0.001 (‘***’).

To better understand the dynamics of pneumococcal lineages and serotypes over time, the study period was divided into three distinct phases. As shown in Fig. 6, there was a significant decrease in the prevalence of serotypes 19F (*P*=0.04) and 9V (*P*=0.002) from 19% and 16% in period 1 (2012–2015) to 5% and 0% in period 3 (2020–2022), respectively. This was associated with a slight decrease in the prevalence of GPSC-44 and GPSC-6 from 16% and 26% during 2012–2015 to 5% and 17% during 2020–2022, respectively. Conversely, serotype 19A experienced a significant increase (*P*<0.001), rising from undetectable levels in period 1–27% in period 3. This was associated with a significant increase in detection rates of GPSC-10 (*P*=0.005) from 2% in 2012–2015 to 22% in 2020–2022. Meanwhile, other dominant serotypes, such as 14, 3 and 6A remained prevalent throughout all study periods.

In addition to shifts in overall prevalence, the serotype composition within certain GPSCs also changed over time (Fig. S4). For instance, the proportion of the non-PCV serotype 9 N increased within GPSC-16 since 2016–2019, partially replacing the PCV serotype 23F. Within GPSC-6, the proportion of PCV serotype 9V declined progressively and became undetected by 2020–2022, while PCV serotype 14 remained relatively stable within the same GPSC.

### Dynamics of AMR over time

**Fig. 7.**
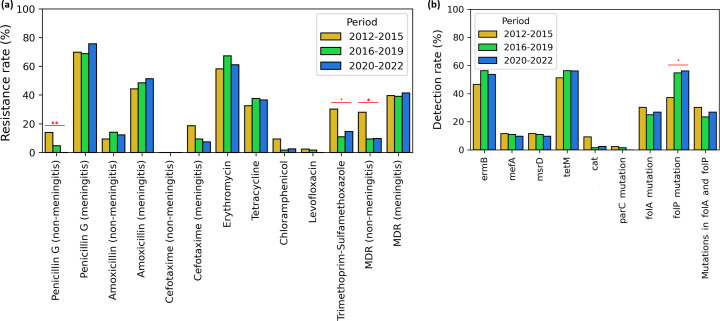
Changes in phenotypic AMR (**a**) and detection rates of AMR determinants (**b**) in *S. pneumoniae* in south Tunisia during 2012–2022. Statistical significance is indicated in red, with the following thresholds: *P*-value between 0.1 and 0.05 (‘.’), *P*-value between 0.05 and 0.01 (‘*’), *P*-value between 0.01 and 0.001 (‘**’) and *P*-value below 0.001 (‘***’).

The temporal analysis of AMR from 2012 to 2022 revealed variations in resistance patterns for beta-lactams and trimethoprim–sulfamethoxazole (Fig. 7a). Based on non-meningitis breakpoints, resistance to penicillin G significantly declined from 14% in 2012–2015 to undetected levels in 2020–2022 (*P*=0.007), coinciding with a significant reduction in MDR rates from 28% to 10% during the same period (*P*=0.018). Using meningitis breakpoints, amoxicillin resistance increased slightly from 44% to 51%, while cefotaxime resistance decreased moderately from 19% to 7%. However, MDR rates under these breakpoints remained stable at around 40%. Resistance to trimethoprim–sulfamethoxazole decreased from 30% in 2012–2015 to 11–15% in 2016–2022 (*P*=0.057). Nevertheless, nonsusceptibility rates (I+R) for this antibiotic showed minimal change, following more closely the trends of the co-detection of *fol*A and *fol*P genes (Fig. 7b). For other antibiotics, there was no notable change in phenotypic resistance rates or AMR determinant detection rates across the 2012–2022 period.

## Discussion

IPD remains a significant public health challenge worldwide. Monitoring the changing patterns of pneumococcal serotypes and their associated genetic characteristics is crucial for guiding effective vaccination and therapeutic strategies. In this study, we provide a comprehensive analysis of the serotype distribution, clonal relationships and antimicrobial resistance determinants of IPD isolates in south Tunisia.

To achieve this, we employed WGS, which offers higher resolution than conventional genotyping methods like pulsed-field gel electrophoresis and multilocus variable-number tandem repeat analysis. WGS enables comprehensive screening of antimicrobial resistance determinants, surpassing classical PCR techniques, which typically detect a limited number of genes per reaction. Additionally, there are multiple WGS-based tools that can predict pneumococcal serotypes [28,38, 39]. Indeed, our findings demonstrated a high concordance between conventional serotyping and *in silico* serotyping methods, with agreement rates comparable to those reported in the literature [40,41]. Moreover, WGS enabled the identification of molecular types 6E(6A) and 6E(6B), which are serologically similar to 6A and 6B, respectively, and cannot be distinguished through conventional techniques.

The distribution of serotypes varies depending on geographic region, patient age, infection type and time [42]. In our study, 26 serotypes were identified using WGS, with serotype 14 being the most common, followed by serotype 3. Serotype 14 has historically been dominant in multiple countries prior to the introduction of PCVs [43]. Serotype 3 is frequently observed in IPD but is much less frequent in carriage cases. A recent Tunisian study revealed that serotype 3 carriage is rare among children under 5 years old [19]. However, it is noteworthy that serotype 1, one of the most common serotypes responsible for IPD globally, and particularly prevalent in African countries [44], was not detected in this study. The absence of serotype 1 in our cohort reflects regional differences in serotype distribution.

Regarding antimicrobial resistance, low penicillin G and amoxicillin resistance (R) rates were observed in our cohort based on non-meningitis breakpoints, though this rate was much higher using meningitis breakpoints. This may be attributed to the high diversity of PBP alleles. Nevertheless, most strains remained susceptible to cefotaxime, supporting third-generation cephalosporins as a suitable treatment option for IPD, particularly in meningitis cases.

ML-based beta-lactam resistance prediction, relying on PBP allele combinations [32,45], demonstrated high specificity in predicting susceptibility (S), as most strains predicted to be susceptible were also phenotypically susceptible. However, there was moderate agreement between WGS and phenotypic methods for beta-lactam ‘susceptibility at increased exposure’ (intermediate, I) and for resistance (R), underscoring the need to update ML models by incorporating recently identified PBP alleles. This improvement is particularly relevant for under-sequenced regions like Tunisia, where a considerable proportion of strains harboured new PBP alleles.

For other antibiotics, macrolide resistance was the most prevalent, aligning with existing literature [46]. This resistance is predominantly driven by the *erm*B gene, which encodes an enzyme that methylates 23S rRNA, leading to target-site modification and resulting in high levels of macrolide resistance. In contrast, the *mef*A gene, which encodes macrolide efflux pumps and contributes to lower levels of macrolide resistance, was found to be ten times less frequent [47]. The high prevalence of the *erm*B-mediated resistance mechanism observed in our study is also reported in European countries, whereas efflux pump mechanisms are more common in North America and Asia [47]. Notably, most isolates carrying the *ermB* gene also harboured the tetracycline resistance gene *tetM*. Previous reports showed that *tetM* is commonly associated with mobile elements, such as conjugative transposons, which frequently carry other resistance genes like *ermB* [48].

Concerning fluoroquinolones, which are commonly used to treat pneumococcal infections alongside beta-lactams and macrolides, resistance is acquired through three main mechanisms: the accumulation of genomic mutations, increased drug efflux and the acquisition of plasmid-encoded resistance genes [49]. In this study, fluoroquinolone resistance rates were low, likely due to more restricted antibiotic usage. The two levofloxacin-resistant cases were linked to mutations in the *par*C gene. The observed AMR rates are mostly consistent with those reported in a recent national study spanning eight hospital centres and over 4,000 clinical isolates [50].

The overall agreement between phenotypic and WGS-based predictions in this study was high across most antibiotics, which reflects the potential of WGS for predicting antimicrobial nonsusceptibility. Notably, most strains lacking a resistance determinant for any antibiotic were phenotypically susceptible to it, affirming the method’s reliability for identifying susceptible strains. Discordant cases where susceptible phenotypes were associated with AMR determinants may be due to the non-expression or under-expression of these resistance genes.

From a genomic perspective, our study revealed a high level of genetic diversity, as indicated by the large number of identified STs. This finding aligns with observations from studies conducted in various countries, where high ST diversity has been reported [51,54]. Several factors contribute to the genetic make-up of *S. pneumoniae*, including the emergence of new clones, mutations and selective pressures imposed by vaccination and antibiotic use.

In recent years, the GPSC classification scheme has gained prominence for grouping pneumococcal isolates into different lineages. This classification system facilitates the tracking of specific clones and their potential public health implications. Within our cohort, the most prevalent GPSCs were GPSC-10, GPSC-6 and GPSC-44, which are all classified as major clusters according to the GPSC database. These clusters are associated with various antibiotic resistance determinants and high MDR rates, making their monitoring crucial. In contrast, other GPSCs such as GPSC-12 and GPSC-83 were largely susceptible to most antibiotics. These two GPSCs are associated with serotype 3, which was previously reported to be susceptible to most antibiotics [55,56].

Literature data indicate that the distribution of GPSCs varies significantly across countries. The two most predominant GPSCs in this study (GPSC-10 and GPSC-6) were also common in countries with strong travel connections to Tunisia, such as Morocco and France, as reported in the GPS database [21]. This suggests potential cross-border transmission, emphasizing the need for regional surveillance. However, these GPSCs were less prevalent in several other African countries [52,57]. This variability in GPSC distribution can be explained by several factors including local epidemiology, environmental conditions, antibiotic usage and vaccination strategies such as the valency and coverage of pneumococcal vaccines. These vaccination efforts, particularly with PCVs, are primarily aimed at reducing the incidence of IPD by targeting the serotypes most commonly associated with severe infections.

In Tunisia, pneumococcal lineage and serotype distribution have shifted notably since PCV7 introduction in the private sector during 2008, followed by PCV10 (GSK) and PCV13 in 2012. Despite limited access, these vaccines initially reduced the prevalence of some vaccine serotypes, such as 19F and 9V. Similar shifts in serotype distribution were also reported in another multicentric Tunisian study involving 903 isolates collected from 2012 to 2019, based on conventional serotyping methods [50]. A major shift occurred in 2019 with the implementation of PCV10 (GSK) in the national immunization programme, leading to broader coverage. This vaccination strategy resulted in significant changes such as the replacement of serotypes 19F and 9V by 19A, associated with a sharp rise in the prevalence of GPSC-10. The increase of serotype 19A following low-valency PCVs vaccination that does not include it has been previously reported in other countries, including Australia, France, Finland [43] and Brazil [58]. Despite vaccination efforts, several pneumococcal lineages, such as GPSC-6, GPSC-12 and GPSC-83, remained predominant throughout the study period. GPSC-12 and GPSC-83 are associated with serotype 3, which is not covered by PCV10 (GSK) and is known to resist vaccination efforts by higher valency PCVs. This low response to vaccination is attributed to mechanisms such as the profuse production and shedding of its capsule, which can overwhelm the protective capacity of antibodies [59]. The prevalence of GPSC-6, however, slightly decreased due to the reduction of the vaccine serotype 9V, while vaccine serotype 14 within GPSC-6 was largely unaffected by vaccination. This suggests that GPSC-6 as a whole is not resistant to vaccination and that few genetic factors may contribute to the persistence of GPSC-6 serotype 14. Nevertheless, a slight decreasing trend in the overall prevalence of serotype 14 was observed in a national multi-centric study during 2012**–**2019 [50], suggesting that vaccination against this serotype is likely still effective. Further investigation is warranted to clarify whether this national decline reflects a reduction of serotype 14 within GPSC-6 or declines across other genetic lineages harbouring this serotype.

This study has some limitations, mainly related to the sampling strategy. The analysis was only based on isolated collections from the microbiology laboratory of Habib Bourguiba University Hospital, which is the main reference laboratory for microbiological diagnostics in the southern part of the country. This laboratory receives specimens from the two largest tertiary hospitals in the region: Hedi Chaker and Habib Bourguiba (~1,600 beds), as well as other secondary healthcare centres. Therefore, our dataset likely provides a reasonably representative overview of pneumococcal serotypes and lineages circulating in south Tunisia. However, the moderate number of IPD isolates sequenced each year limits the capacity to detect minor changes in serotype and lineage distribution.

Overall, this study represents the first comprehensive genomic characterization of *S. pneumoniae* in Tunisia. Our findings revealed changes in the serotype distribution and the genomic landscape from 2012 to 2022, following vaccination efforts. This led to a decrease in the prevalence of several GPSCs associated with vaccine serotypes. Nevertheless, the emergence of GPSC-10, characterized by high antimicrobial resistance levels, highlights the need for continuous genomic surveillance. Additional strategies to address this ongoing challenge are required, such as antibiotic stewardship programmes and the implementation of PCVs targeting serotype 19A, which is the dominant serotype within GPSC-10.

## Supplementary material

10.1099/mgen.0.001448 Uncited Supplementary Material 1.

## Abbreviations

AMR: antimicrobial resistance
CC: clonal complex
GPS: Global Pneumococcal Sequencing
GPSC: Global Pneumococcal Sequence Cluster
IPD: invasive pneumococcal disease
MDR: multi-drug resistance
MIC: minimum inhibitory concentration
ML: machine learning
PBP: penicillin-binding protein
PCV: pneumococcal conjugate vaccine
ST: sequence type
WGS: whole-genome sequencing
