# Supplementary material for: Genomic surveillance of invasive Streptococcus pneumoniae strains in south Tunisia during 2012–2022
Source: Microb Genom. 2025 Jul 17;11(7):001448. doi: 10.1099/mgen.0.001448 (PMC12453412; doi:10.1099/mgen.0.001448)
Supplement: Uncited Supplementary Material 1. [file mgen-11-01448-s001.pdf]

Table S1. Accession numbers of invasive *S. pneumoniae* sequences included in this study

Sample ID	BioSample Accession no.	SRA Accession no.	Genbank Accession no.	Collection date
A48-16	SAMN44581123	SRS23108105	JBOIUH000000000	29/01/2016
A953-12	SAMN44581124	SRS23108104	JBOIUI000000000	31/12/2012
B118-14	SAMN44581125	SRS23108117	JBOIUJ000000000	21/01/2014
B1354-13	SAMN44581126	SRS23108129	JBOIUK000000000	07/05/2013
B1523-17	SAMN44581127	SRS23108140	JBOIUL000000000	23/08/2017
B1563-19	SAMN44581128	SRS23108150	JBOIUM000000000	04/08/2019
B1756-16	SAMN44581129	SRS23108161	JBOIUN000000000	05/08/2016
B1775-18	SAMN44581130	SRS23108172	JBOIUO000000000	04/10/2018
B1977-13	SAMN44581131	SRS23108183	JBOIUP000000000	03/07/2013
B200-22	SAMN44581132	SRS23108188	JBOIUQ000000000	27/01/2022
B2130-21	SAMN44581133	SRS23108106	JBOIUR000000000	03/12/2021
B2167-14	SAMN44581134	SRS23108108	JBOIUS000000000	18/11/2014
B2203-16	SAMN44581135	SRS23108109	JBOIUT000000000	05/10/2016
B2507-22	SAMN44581136	SRS23108110	JBOIUU000000000	02/11/2022
B254-22	SAMN44581137	SRS23108111	JBOIUV000000000	01/02/2022
B272-19	SAMN44581138	SRS23108112	JBOIUW000000000	25/02/2019
B2994-22	SAMN44581139	SRS23108113	JBOIUX000000000	25/12/2022
B303-20	SAMN44581140	SRS23108114	JBOIUY000000000	13/02/2020
B3058-15	SAMN44581141	SRS23108115	JBOIUZ000000000	03/12/2015
B317-19	SAMN44581142	SRS23108116	JBOIVA000000000	01/03/2019
B357-19	SAMN44581143	SRS23108118	JBOIVB000000000	03/03/2019
B36-21	SAMN44581144	SRS23108119	JBOIVC000000000	11/01/2021
B371-19	SAMN44581145	SRS23108120	JBOIVD000000000	08/03/2019
B407-16	SAMN44581146	SRS23108121	JBOIVE000000000	24/02/2016
B451-18	SAMN44581147	SRS23108122	JBOIVF000000000	30/03/2018

B485-19	SAMN44581148	SRS23108123	JBOIVG000000000	27/03/2019
B487-21	SAMN44581149	SRS23108124	JBOIVH000000000	16/04/2021
B499-19	SAMN44581150	SRS23108125	JBOIVI000000000	27/03/2019
B539-12	SAMN44581151	SRS23108126	JBOIVJ000000000	13/02/2012
B565-13	SAMN44581152	SRS23108127	JBOIVK000000000	21/02/2013
B726-13	SAMN44581153	SRS23108128	JBOIVL000000000	08/03/2013
B810-13	SAMN44581154	SRS23108130	JBOIVM000000000	18/03/2013
B902-16	SAMN44581155	SRS23108131	JBOIVN000000000	24/04/2016
C1000-22	SAMN44581156	SRS23108132	JBOIVO000000000	28/05/2022
C113-20	SAMN44581157	SRS23108133	JBOIVP000000000	16/01/2020
C1398-20	SAMN44581158	SRS23108134	JBOIVQ000000000	24/11/2020
C1458-20	SAMN44581159	SRS23108135	JBOIVR000000000	26/12/2020
C2351-19	SAMN44581160	SRS23108136	JBOIVS000000000	19/09/2019
C2626-19	SAMN44581161	SRS23108137	JBOIVT000000000	11/10/2019
C2743-18	SAMN44581162	SRS23108138	JBOIVU000000000	07/11/2018
C289-18	SAMN44581163	SRS23108139	JBOIVV000000000	07/02/2018
C3432-16	SAMN44581164	SRS23108141	JBOIVW000000000	22/10/2016
C3877-14	SAMN44581165	SRS23108142	JBOIVX000000000	31/12/2014
C412-14	SAMN44581166	SRS23108143	JBOIVY000000000	16/02/2014
C4229-16	SAMN44581167	SRS23108144	JBOIVZ000000000	20/12/2016
C457-20	SAMN44581168	SRS23108145	JBOIWA000000000	25/02/2020
C474-13	SAMN44581169	SRS23108146	JBOIWB000000000	11/02/2013
C566-14	SAMN44581170	SRS23108147	JBOIWC000000000	03/03/2014
C633-13	SAMN44581171	SRS23108148	JBOIWD000000000	25/02/2013
C765-19	SAMN44581172	SRS23108149	JBOIWE000000000	01/04/2019
C815-22	SAMN44581173	SRS23108151	JBOIWF000000000	18/05/2022
CR262-16	SAMN44581174	SRS23108152	JBOIWG000000000	20/07/2016

CR96-18	SAMN44581175	SRS23108153	JBOIWH000000000	30/03/2018
CR99-12	SAMN44581176	SRS23108154	JBOIWI000000000	02/03/2012
ED433-16	SAMN44581177	SRS23108155	JBOIWJ000000000	08/04/2016
F143-16	SAMN44581178	SRS23108156	JBOIWK000000000	15/12/2016
G283-19	SAMN44581179	SRS23108157	JBOIWL000000000	08/06/2019
H820-16	SAMN44581180	SRS23108158	JBOIWM000000000	19/10/2016
I11-18	SAMN44581181	SRS23108159	JBOIWN000000000	03/01/2018
I158-15	SAMN44581182	SRS23108160	JBOIWO000000000	28/02/2015
I647-22	SAMN44581183	SRS23108162	JBOIWP000000000	14/11/2022
I775-15	SAMN44581184	SRS23108163	JBOIWQ000000000	23/11/2015
J155-22	SAMN44581185	SRS23108164	JBOIWR000000000	14/06/2022
J49-18	SAMN44581186	SRS23108165	JBOIWS000000000	20/02/2018
L164-15	SAMN44581187	SRS23108166	JBOIWT000000000	18/04/2015
L340-18	SAMN44581188	SRS23108167	JBOIWU000000000	14/08/2018
L519-14	SAMN44581189	SRS23108168	JBOIWV000000000	11/12/2014
N1936-21	SAMN44581190	SRS23108169	JBOIWW000000000	22/12/2021
N586-15	SAMN44581191	SRS23108170	JBOIWX000000000	21/03/2015
NP116-12	SAMN44581192	SRS23108171	JBOIWY000000000	02/02/2012
NP362-16	SAMN44581193	SRS23108173	JBOIWZ000000000	23/03/2016
NP402-22	SAMN44581194	SRS23108174	JBOIXA000000000	22/04/2022
O108-19	SAMN44581195	SRS23108175	JBOIXB000000000	22/03/2019
O383-19	SAMN44581196	SRS23108176	JBOIXC000000000	07/12/2019
O507-15	SAMN44581197	SRS23108177	JBOIXD000000000	05/12/2015
P1147-22	SAMN44772815	SRS23228517	JBOIXE000000000	31/10/2022
P12-18	SAMN44581198	SRS23108178	JBOIXF000000000	06/01/2018
P210-21	SAMN44581199	SRS23108179	JBOIXG000000000	03/07/2021
P221-18	SAMN44581200	SRS23108180	JBOIXH000000000	05/03/2018

P22-16	SAMN44581201	SRS23108181	JBOIXI000000000	09/01/2016
P307-18	SAMN44581202	SRS23108182	JBOIXJ000000000	28/03/2018
P919-19	SAMN44772816	SRS23228518	JBOIXK000000000	28/12/2019
PUR1272-22	SAMN44772817	SRS23228529	JBOIXL000000000	28/06/2022
PUR1342-19	SAMN44772818	SRS23228540	JBOIXM000000000	23/05/2019
PUR138-14	SAMN44772819	SRS23228563	JBOIXN000000000	05/02/2014
PUR189-16	SAMN44772820	SRS23228574	JBOIXO000000000	28/01/2016
PUR1897-21	SAMN44772821	SRS23228549	JBOIXP000000000	10/11/2021
PUR2079-15	SAMN44772822	SRS23228553	JBOIXQ000000000	23/12/2015
PUR2819-19	SAMN44581203	SRS23108204	JBOIXR000000000	03/10/2019
PUR311-13	SAMN44772823	SRS23228554	JBOIXS000000000	28/02/2013
PUR3196-17	SAMN44772824	SRS23228555	JBOIXT000000000	30/11/2017
PUR3321-19	SAMN44581204	SRS23108203	JBOIXU000000000	22/11/2019
PUR396-19	SAMN44772825	SRS23228519	JBOIXV000000000	14/02/2019
PUR447-20	SAMN44581205	SRS23108202	JBOIXW000000000	08/02/2020
PUR475-22	SAMN44772826	SRS23228520	JBOIXX000000000	23/03/2022
PUR548-18	SAMN44772827	SRS23228521	JBOIXY000000000	07/03/2018
PUR683-22	SAMN44772828	SRS23228522	JBOIXZ000000000	22/04/2022
PUR772-13	SAMN44772829	SRS23228523	JBOIYA000000000	05/06/2013
PUR790-16	SAMN44772830	SRS23228524	JBOIYB000000000	11/04/2016
PUR87-15	SAMN44772831	SRS23228525	JBOIYC000000000	19/01/2015
PUR943-14	SAMN44772832	SRS23228526	JBOIYD000000000	15/07/2014
PY03-20	SAMN44772833	SRS23228527	JBOIYE000000000	03/01/2020
PY10-22	SAMN44772834	SRS23228528	JBOIYF000000000	10/02/2022
PY116-19	SAMN44772835	SRS23228530	JBOIYG000000000	28/10/2019
PY119-22	SAMN44772836	SRS23228531	JBOIYH000000000	05/12/2022
PY18-16	SAMN44772837	SRS23228532	JBOIYI000000000	10/03/2016

PY46-20	SAMN44772838	SRS23228533	JBOIYJ000000000	16/07/2020
PY49-13	SAMN44772839	SRS23228534	JBOIYK000000000	01/04/2013
PY49-19	SAMN44772840	SRS23228535	JBOIYL000000000	03/04/2019
PY52-16	SAMN44581206	SRS23108184	JBOIYM000000000	26/05/2016
PY57-14	SAMN44772841	SRS23228536	JBOIYN000000000	08/07/2014
PY70-22	SAMN44772843	SRS23228538	JBOIYO000000000	13/07/2022
PY7-21	SAMN44772842	SRS23228537	JBOIYP000000000	22/01/2021
PY76-12	SAMN44772844	SRS23228539	JBOIYQ000000000	21/08/2012
PY91-21	SAMN44772845	SRS23228541	JBOIYR000000000	29/11/2021
PY93-21	SAMN44772846	SRS23228542	JBOIYS000000000	09/12/2021
PY98-14	SAMN44772847	SRS23228543	JBOIYT000000000	24/10/2014
R612-22	SAMN44581207	SRS23108201	JBOIYU000000000	25/02/2022
RH177-16	SAMN44772848	SRS23228556	JBOIYV000000000	30/05/2016
RM772-21	SAMN44772849	SRS23228557	JBOIYW000000000	29/10/2021
S103-13	SAMN44772850	SRS23228558	JBOIYX000000000	28/01/2013
S1106-17	SAMN44772851	SRS23228559	JBOIYY000000000	23/12/2017
S124-13	SAMN44772852	SRS23228560	JBOIYZ000000000	02/02/2013
S183-19	SAMN44772853	SRS23228561	JBOIZA000000000	10/03/2019
S2-19	SAMN44772854	SRS23228562	JBOIZB000000000	02/01/2019
S237-19	SAMN44772855	SRS23228564	JBOIZC000000000	23/03/2019
S255-21	SAMN44772856	SRS23228565	JBOIZD000000000	11/04/2021
S327-19	SAMN44772857	SRS23228567	JBOIZE000000000	14/05/2019
S396-13	SAMN44772858	SRS23228566	JBOIZF000000000	22/04/2013
S402-19	SAMN44772859	SRS23228568	JBOIZG000000000	24/06/2019
S418-13	SAMN44772860	SRS23228569	JBOIZH000000000	29/04/2013
S433-18	SAMN44772861	SRS23228571	JBOIZI000000000	26/05/2018
S481-17	SAMN44772862	SRS23228570	JBOIZJ000000000	06/06/2017

S504-20	SAMN44772863	SRS23228572	JBOIZK000000000	09/10/2020
S53-14	SAMN44772864	SRS23228573	JBOIZL000000000	19/01/2014
S545-22	SAMN44772865	SRS23228576	JBOIZM000000000	07/09/2022
S600-18	SAMN44772866	SRS23228575	JBOIZN000000000	13/07/2018
S767-12	SAMN44772867	SRS23228577	JBOIZO000000000	11/08/2012
S790-21	SAMN44772868	SRS23228578	JBOIZP000000000	13/12/2021
S844-14	SAMN44772869	SRS23228579	JBOIZQ000000000	10/07/2014
S954-16	SAMN44772870	SRS23228544	JBOIZR000000000	31/10/2016
S96-17	SAMN44772871	SRS23228545	JBOIZS000000000	02/02/2017
UB55-12	SAMN44772872	SRS23228546	JBOIZT000000000	13/03/2012
Z793-15	SAMN44772873	SRS23228547	JBOIZU000000000	08/12/2015
Z829-22	SAMN44772874	SRS23228548	JBOIZV000000000	26/10/2022
ZB104-17	SAMN44772875	SRS23228550	JBOIZW000000000	27/04/2017
ZB140-15	SAMN44772876	SRS23228551	JBOIZX000000000	15/05/2015
ZB30-22	SAMN44772877	SRS23228552	JBOIZY000000000	01/02/2022

Table S2. Beta-lactam MIC breakpoints according to the recommendations of the Antibiogram Committee of the French Society of Microbiology which is aligned with the guidelines of the European Committee on Antimicrobial Susceptibility Testing (CA-SFM/EUCAST, 2021).

**Antibiotic**	**Non-meningitis Susceptibility Breakpoints (mg/L) (S ≤)**	**Non-meningitis Resistance Breakpoints (mg/L) (R >)**	**Meningitis Resistance Breakpoints (mg/L) (R >)**
Penicillin G	0,064	2	0,064
Amoxicillin/Ampicillin	0.5	2	0.5
Cefotaxime	0.5	2	0.5

Table S3. Phenotypic and WGS-based typing results for *S. pneumoniae* strains with serotype discordances

Sample ID	Phenotypic serotype	Serotype (WGS)	GPSC	ST
S418-13	09A	09V	6	6521
B1977-13	06B	6E(6A)	47	16104
S844-14	09A	09V	6	6521
B2203-16	18C	18B	67	1381
H820-16	18C	18A	95	241
C457-20	06B	06A	9	2105

Table S4. Allelic profiles of the newly identified sequence types (STs)

ST	*aro*E	*gdh*	*gki*	*rec*P	*spi*	*xpt*	*ddl*
ST-19892	697*	11	34	16	15	1	145
ST-19893	7	5	4	5	42	1	14
ST-19894	32	28	1	644*	15	52	14
ST-19895	7	5	4	5	863*	1	18
ST-19896	5	25	4	4	15	20	28
ST-19897	7	9	4	1	10	6	26

**New allele*

Table S5. Distribution of sequenced invasive *S. pneumonaie* isolates according to serotypes, GPSCs and ST between 2012 and 2022

Serotype*	GPSC**	ST***
14 (26)	6 (25), 9 (1)	2918 (16), 156 (1), 63 (1), 143 (5), 4949 (1), 4344 (1), 8995 (1)
3 (19)	12 (12), 83 (7)	180 (8), 505 (4), 1220 (3), 260 (3), 16168 (1)
19A (18)	10 (15), 5 (1), 53 (1), 1 (1)	3772 (14), 276 (1), 17471 (1), 847 (1), 320 (1)
19F (16)	44 (15), 10 (1)	179 (14), 2307 (1), 16235 (1)
09V (9)	6 (7), 43 (2)	156 (3), 838 (2), 280 (2), 6521 (2)
06A (9)	13 (5), 9 (3), 213 (1)	2105 (3), 473 (2), 19895[##] (1), 19896[##] (1), 16105 (1), 5679 (1)
6E(6B) (8)	47 (7), 185 (1)	386 (6), 19894[##] (1), 16106 (1)
23F (7)	16 (5), 9 (2)	4003 (3), 81 (2), 63 (2)
18C (5)	67 (4), 43 (1)	1233 (2), 280 (1), 19892[##] (1), 1381 (1)
09N (6)	16 (5), 699 (1)	8809 (4), 517 (1), 6359 (1)
4 (4)	70 (2), 27 (2)	1221 (2), 205 (2)
35B (3)	59 (3)	558 (3)
16F (2)	156 (2)	3551 (1), 2685 (1)
07F (2)	32 (1), 15 (1)	3544 (1), 191 (1)
35F (2)	126 (2)	19893[##] (2)
13 (1)	323 (1)	2187 (1)
6E(6A) (1)	47 (1)	16104 (1)
07C (1)	18 (1)	1201 (1)
18B (1)	67 (1)	1381 (1)
18A (1)	95 (1)	241 (1)
17F (1)	49 (1)	13010 (1)
Serogroup 24 (1)	10 (1)	4253 (1)
5 (1)	8 (1)	289 (1)
23A (1)	5 (1)	10272 (1)
34 (1)	1124[#] (1)	19897[##] (1)
8 (1)	277 (1)	6197 (1)
11A (1)	3 (1)	62 (1)

**Serotype determined using the in-silico typing tool "SeroBA" based on whole-genome sequencing data*

***GPSC: Global Pneumococcal Sequence Cluster*

****ST: Sequence type*

[#]New GPSC

[##]New ST

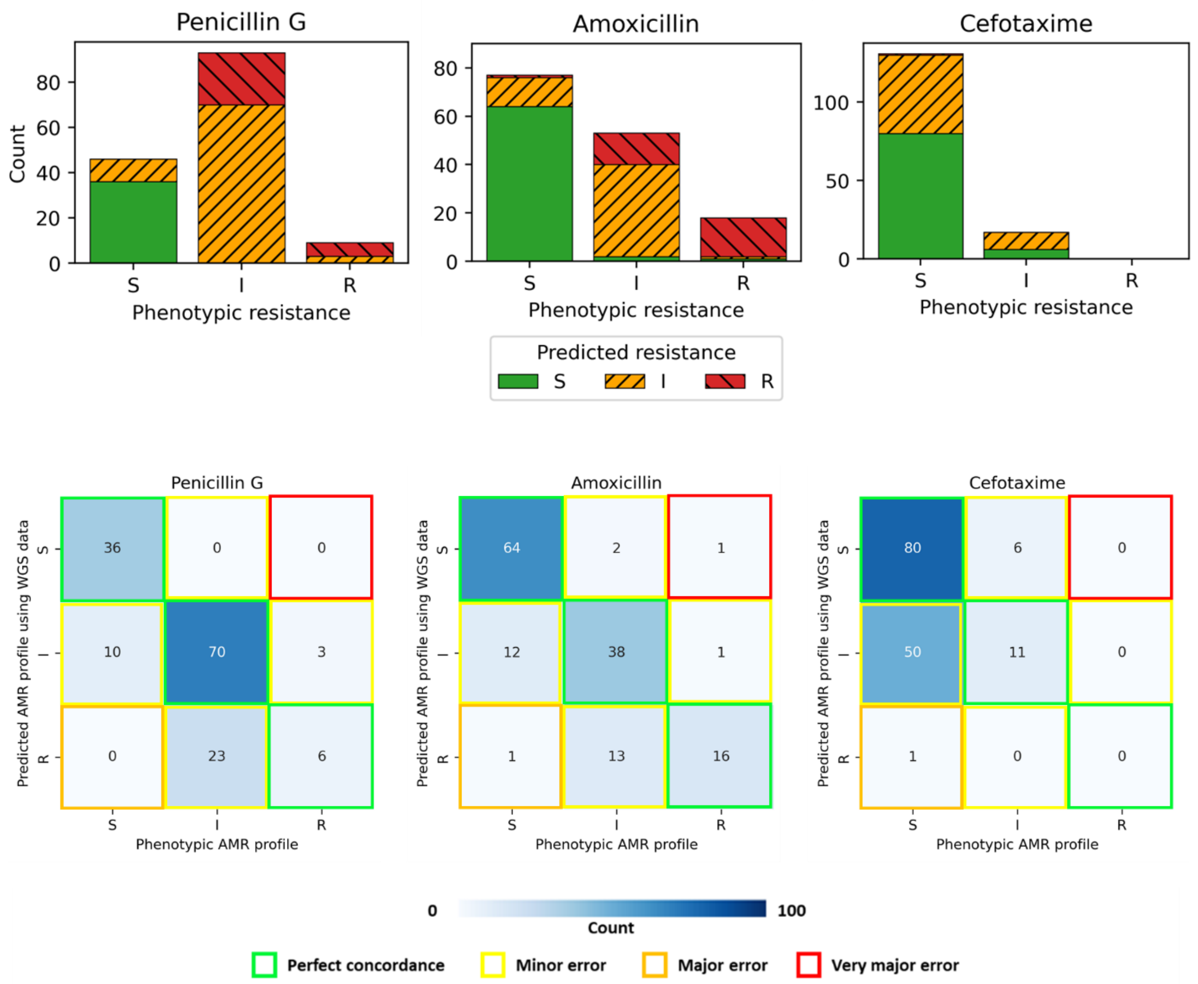

Fig. S1. Concordance analysis between phenotypic beta-lactams resistance compared to WGS-based machine learning predictions

S: susceptible, standard dosing regimen; I: susceptible, increased exposure (intermediate); R: resistant. This analysis was performed based on non-meningitis breakpoints of each beta-lactam. All I+R cases are considered as R when considering meningitis breakpoints

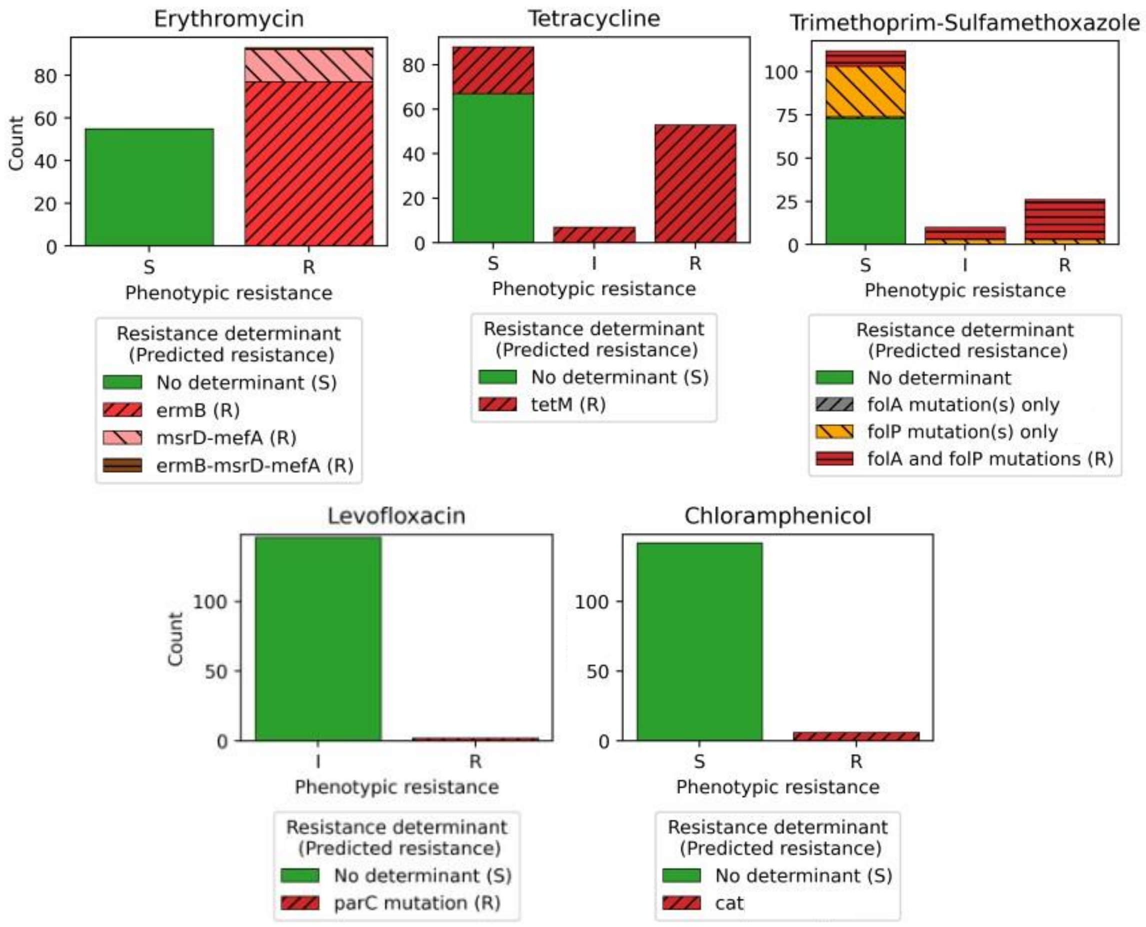

Fig. S2. Distribution of resistance determinants according to phenotypic resistance of the main tested antibiotics

S: susceptible, standard dosing regimen; I: susceptible, increased exposure (intermediate); R: resistant.

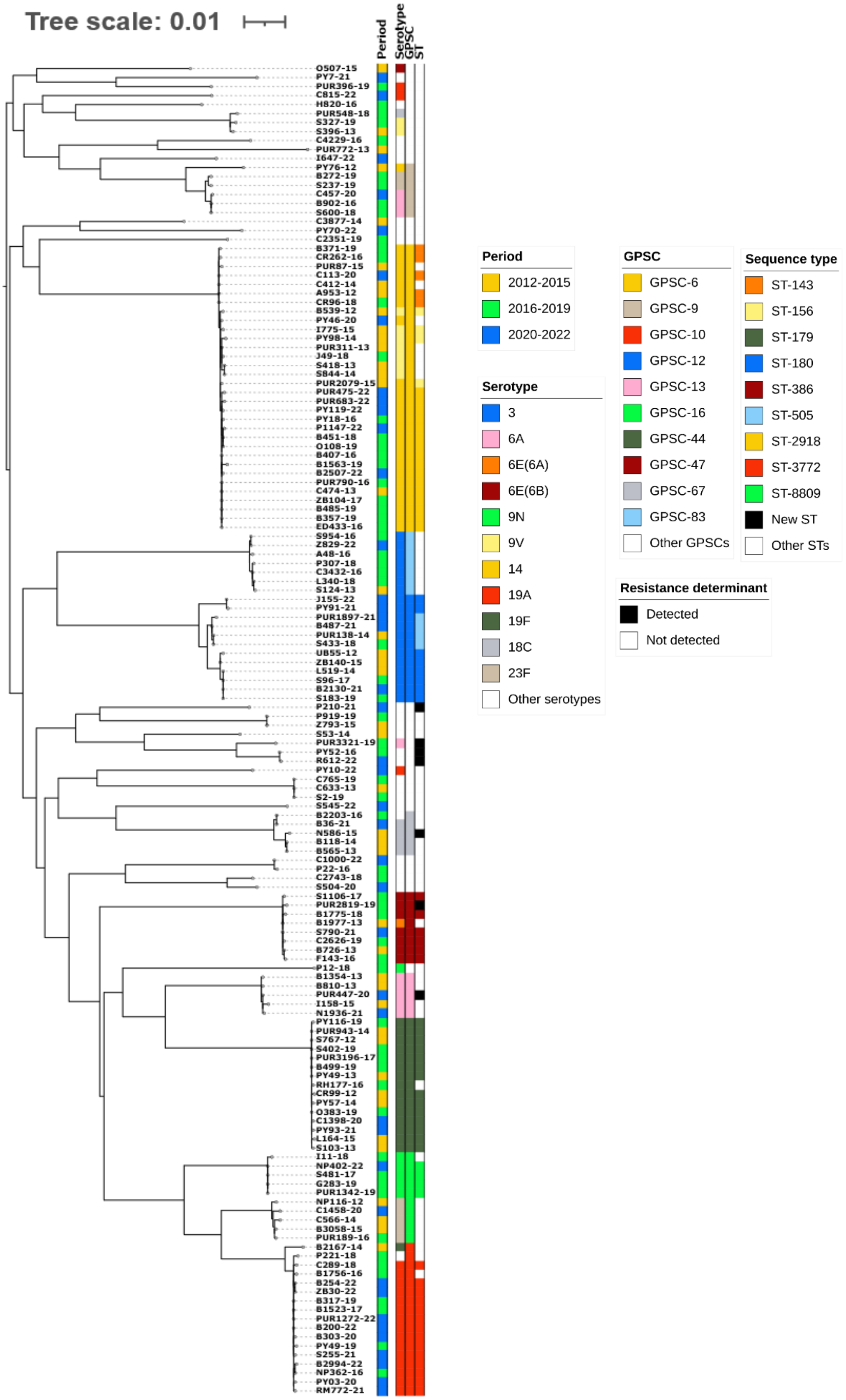

Fig. S3. Rectangular phylogenetic tree of the 148 *S. pneumoniae* isolates responsible for invasive pneumococcal diseases (South Tunisia, 2012–2022)

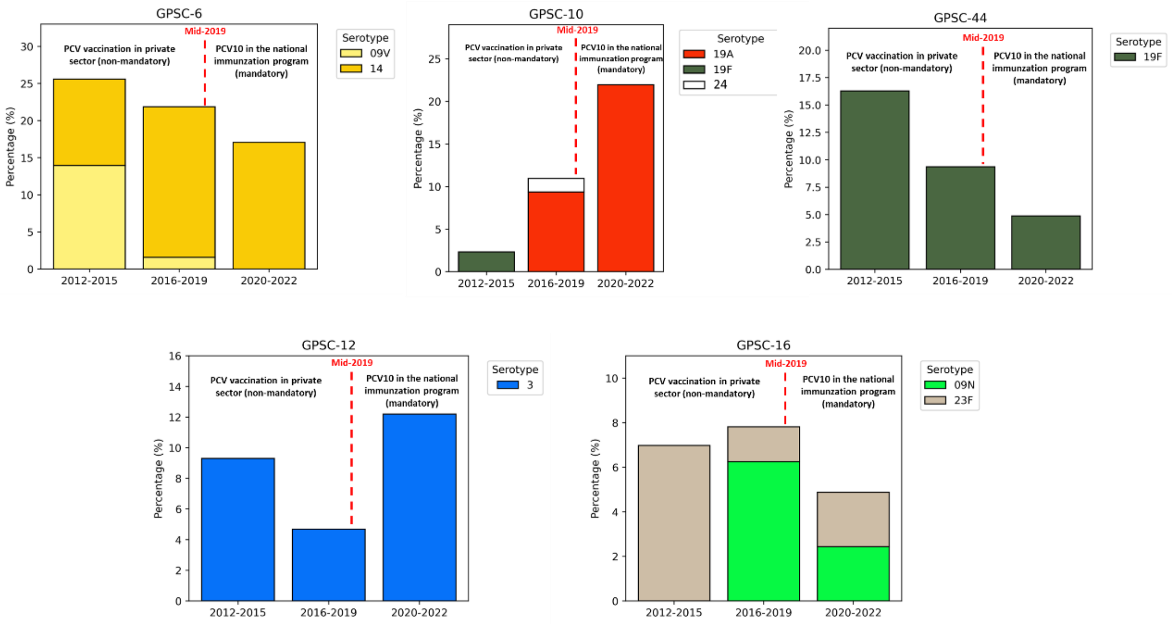

Fig. S4. Changes in serotype distribution within the five major *S. pneumoniae* GPSCs in south Tunisia over time (2012–2022)

The color scheme matches that of the phylogenetic tree to facilitate visual comparison.
